# Assessment of Food Safety Knowledge, Attitudes and Practices of Food Service Staff in Bangladeshi Hospitals: A Cross-Sectional Study

**DOI:** 10.3390/nu14122540

**Published:** 2022-06-18

**Authors:** Md. Hasan Al Banna, Md Shafiqul Islam Khan, Humayra Rezyona, Abdul-Aziz Seidu, Mohammad Tazrian Abid, Tasnu Ara, Satyajit Kundu, Bright Opoku Ahinkorah, John Elvis Hagan, Jr., Md. Abu Tareq, Musammet Rasheda Begum, Mohammad Faizul Tawhid Chowdhury, Thomas Schack

**Affiliations:** 1 Department of Food Microbiology, Faculty of Nutrition and Food Science, Patuakhali Science and Technology University, Patuakhali 8602, Bangladesh; banna.nfs.pstu@gmail.com (M.H.A.B.); msikhan312@yahoo.com (M.S.I.K.); abutareqfmb@pstu.ac.bd (M.A.T.); 2 Department of Food and Nutrition, College of Home Economics, Azimpur, Dhaka 1205, Bangladesh; humayra.rezyona@gmail.com (H.R.); tasnu610@gmail.com (T.A.); 3 Department of Real Estate Management, Takoradi Technical University, Takoradi P.O. Box 256, Ghana; abdul-aziz.seidu@stu.ucc.edu.gh; 4 Centre for Gender and Advocacy, Takoradi Technical University, Takoradi P.O. Box 256, Ghana; 5 College of Public Health, Medical and Veterinary Sciences, James Cook University, Townsville 4811, Australia; 6 Faculty of Nutrition and Food Science, Patuakhali Science and Technology University, Patuakhali 8602, Bangladesh; tazrian32rwam@gmail.com (M.T.A.); or satyajit@seu.edu.cn (S.K.); 7 School of Public Health, Southeast University, Nanjing 210009, China; 8 School of Public Health, Faculty of Health, University of Technology Sydney, Sydney 2007, Australia; brightahinkorah@gmail.com; 9 Department of Health, Physical Education & Recreation, College of Education Studies, University of Cape Coast, Cape Coast PMB TF0494, Ghana; 10 Neurocognition and Action-Biomechanics-Research Group, Faculty of Psychology and Sports Science, Bielefeld University, Postfach 10 01 31, 33501 Bielefeld, Germany; thomas.schack@uni-bielefeld.de; 11 Department of Agricultural Economics and Social Sciences, Chattogram Veterinary and Animal Sciences University, Chattogram 4225, Bangladesh; rasustat@yahoo.com; 12 Junior Consultant (Opthalmology), Sheikh Fazilatunnesa Mujib Eye Hospital and Training Institute, Gopalganj 8100, Bangladesh; tawhidcmc@yahoo.com

**Keywords:** food safety knowledge, attitude, handling practices, food service employees, hospitals, Bangladesh

## Abstract

Food safety knowledge, attitudes and practices among hospital food service staff are crucial in the prevention of foodborne disease outbreaks, as hospitalized patients are more vulnerable to potential hazards. This study, therefore, sought to assess the food safety knowledge, attitudes and practices of food service staff in Bangladeshi hospitals. A cross-sectional study was conducted among 191 food service staff from seven different hospitals in Dhaka and Chattogram from October 2021 to March 2022 using pretested questionnaires. Multiple linear regression was used to identify the factors associated with the food safety knowledge, attitudes and practices. The findings showed moderate knowledge but high levels of attitudes and practices of food safety among hospital food handlers. Food safety knowledge was significantly higher among males, participants from private hospitals and participants working in a hospital that had a food service supervisor and dietitian in charge of food service operations. Moreover, participants from private hospitals and participants working in a hospital that had a food service supervisor and dietitian in charge of food service operations had more positive attitudes and better practices regarding food safety. Hospital management should consider these factors for enhancing food handlers’ knowledge and increase training and supervision on food safety practices to reduce foodborne diseases and outbreaks.

## 1. Introduction

Globally, food safety is a major concern for consumers, food service institutions and regulatory authorities. Every year, millions of people around the world are hospitalized and even death due to the consumption of contaminated food [1]. About 70% of those foodborne illnesses were associated with food service establishments or catering, and the risk of death from hospital outbreaks was estimated to be three times higher than in other settings [2]. Epidemiological and surveillance data show that hospitals are becoming breeding grounds for foodborne diseases [3,4,5]. It is evident that inappropriate practices in food service establishments play a vital role in the causal chain of foodborne diseases. This phenomenon has also been shown to be critical in some nosocomial foodborne outbreaks in hospitals [4,6]. Moreover, hospital kitchens are listed as one of the main sources of contamination [3] that cause outbreaks of foodborne illness by cross-contamination [2]. A study on hospital-acquired listeriosis (i.e., a foodborne disease) was found to be linked to utensils used in hospital kitchens [3]. Meakins et al. [2] reported that 65% of hospital inpatients and 12% of staff were affected by Salmonellosis, and mortality risk was highest in foodborne disease outbreaks. Generally, food safety is more sensitive for hospitalized patients because they are more vulnerable to foodborne diseases than the general population due to low immunity and malnutrition [6], but sometimes less attention is given compared to other institutions [7]. An outbreak of a foodborne disease in hospitals can cause morbidity and mortality for anyone infected, especially the already vulnerable patients, and also cause service disruption leading to increase hospitalization costs [8,9].

In hospitals, food service staff can be a potential source of food contamination and hospital-related foodborne outbreaks, as they may transmit pathogens into foods throughout the chain of food preparation, processing, storage and distribution [6,10]. Serving patients food that is contaminated by poor hygienic practices of food handlers can lead to food poisoning, which can lead to an outbreak throughout the hospital [11,12].

In Bangladesh, the prevalence of foodborne diseases is significantly higher due to overpopulation, underdeveloped infrastructure and poor water, sanitation and hygiene (WASH) conditions [13]. Each year, approximately 30 million people suffer from one form of foodborne disease in Bangladesh [14]. Diarrheal diseases, enteric fever and hepatitis are the top three foodborne diseases in Bangladesh [15]. In 2018, there were approximately 1,122,681 cases of diarrhea at different levels of healthcare facilities in Bangladesh [16], and the country spent $79 million on diarrhea treatment, estimating about 4% of GDP per capita that year [17]. According to the foodborne illness surveillance of the Dhaka-based Institute of Epidemiology, Disease Control and Research (IEDCR), there were an estimated 30,000 and 500 cases of enteric fever and hepatitis in 2015, respectively [18,19]. However, there is no data on foodborne illness outbreaks or food poisoning directly caused due to the food service staff or food handlers of hospitals in Bangladesh.

The health system in Bangladesh has undergone a number of reforms, including extensive health infrastructural development. This infrastructure is pluralistically defined across four key areas: government, the private sector, NGOs and donor agencies, among whom the government, or public sector, is accountable, not only for policy and regulation but also for funding and recruiting health workers [20]. Hospitals in Bangladesh are often overcrowded with patients, family caregivers and visitors, have intermittent water supply and an inadequate number of handwashing stations and toilets [21,22]. A recent study reported that only 15.4% of service receivers were found to be satisfied with the dietary services in a cancer research hospital in Bangladesh [23]. Proper monitoring and resources for infection control in hospitals are severely limited in Bangladesh, which leads to infections in the hospital staff and family caregivers [21,24,25]. To control communicable diseases, the Government of Bangladesh has enacted various acts [20], but the enforcement of food safety laws and regulations in the country is weak due to several limitations within legal and regulatory systems [18]. The Directorate General of Health Services (DGHS) and IEDCR under the Ministry of Health and Family Welfare (MoHFW) are involved in the food safety with its enforcement and surveillance activities that collect information regarding the severity and prevalence of foodborne illness and identify hotspots and definite sources of foodborne pathogens across Bangladesh [18]. However, there is a need to create a framework for collaborative operation agreements between the Bangladesh Food Safety Authority (BFSA), MoHFW, and other food control agencies to take regulatory actions to reduce food safety risks.

Several studies have been done to assess food safety knowledge, attitudes and practices of food handlers in hospitals in different countries such as India [26], Sri Lanka [27], Malaysia [28], Lebanon [29], Sudan [30], Jordan [31], South Africa [32] and Saudi Arabia [33]. These studies reported diversified levels of knowledge, attitudes and practices scores in different countries. Associations were found between food safety knowledge and sociodemographic factors such as age, education level, working experience, previous training courses and type of hospitals in some studies [28,29,33]. Many studies emphasized educational intervention programs or training sessions on food safety knowledge as they commenced a significant positive improvement in food handlers’ knowledge after an intervention [26,29,33]. Moreover, food handlers’ education level and years of experience were associated with food safety practices [29,33].

In Bangladesh, few studies have been conducted to evaluate food safety knowledge, attitudes and practices of meat handlers, chicken vendors, fish farmers, food handlers of baking industries and consumers’ knowledge and awareness of food safety [34,35,36,37,38]. While food safety in hospitals is a great matter of concern, there is a scarcity of published research on the knowledge, attitudes and practices of food service staff in Bangladeshi hospitals. For fostering and enforcing food safety in healthcare facilities, it is imperative to assess the knowledge, attitudes and practices of food handlers in hospitals in Bangladesh. In order to address the gap in the literature, the current study aimed to assess the food safety knowledge, attitudes and practices of food service staff in Bangladeshi hospitals. This study also aims to examine the factors associated with food safety knowledge, attitudes and practices among the sample.

## 2. Methods and Materials

### 2.1. Study Settings

The current survey was conducted on 7 hospitals from two different sectors, private and public. The hospitals were conveniently chosen from Dhaka and Chattogram, two major cities in Bangladesh. In these hospitals, the meals are prepared in the hospitals’ kitchen and/or foods are sold in the cafeteria by food service staff who manage food services for patients, medical staff and visitors. Dietary regimens, patients’ needs and preferences are taken into consideration when plating meals. To ensure appropriate quality standards are maintained, monitoring panels have been appointed by these hospitals to inspect food hygiene and quality aspects of food services systems.

### 2.2. Study Design, Subjects and Sampling

A hospital-based cross-sectional study was carried out from October 2021 to March 2022, aimed at assessing food safety knowledge, attitudes and practices among food service staff in the selected hospitals of Bangladesh. In this study, we defined food service staff as employees who work in the hospital kitchens (e.g., cooks, utensils cleaners and supporting staff), handle food from the kitchen to wards/cabins and work in the cafeteria dining for medical staff and visitors (e.g., storekeepers, waiters and catering staff). The study subjects were included when the following inclusion criteria were fulfilled: (i) food service staff aged 18 years and above and (ii) Bangladeshi by birth. Food service staff who were ill and out of the service during the data collection period were excluded from the study. A purposive sampling technique was applied to select the participants in this study [39]. The study protocols and design were reviewed, revised and approved by the Research Ethical Committee (REC) of the Department of Food Microbiology, Patuakhali Science and Technology University, Bangladesh (approval number: FMB: 13/09/2021:03).

### 2.3. Sample Size Calculation

A sample size of 384 was calculated using Cochran’s formula [34], $n_{o}=\frac{Z^{2}\mathrm{pq}}{e^{2}}$, where n_o_ = Cochran’s sample size recommendation, Z is 1.96 at 95% confidence interval, e is the margin of error at 5% (standard deviation of 0.05) and q = 1 − p. Since there was no previous study on food safety knowledge, attitudes and practices among food service staff working in Bangladeshi hospitals, p = 50% was used. Later, the following modified Cochran’s formula [34] was used for calculating the adjusted sample size in a small population: $n=\;\frac{n_{o}}{1+\;\frac{(n_{o}-1)}{N}}$, here n = adjusted sample size, n_o_ = 384 (Cochran’s sample size recommendation) and N = population size (assuming 1000 food service staff work in hospitals in these study areas). This calculation was given a minimum sample size of 277. We interviewed all the food service staff available on the day of data collection for each hospital. However, this study included a sample of 191 food service staff. Due to the exclusion procedures (i.e., illness and out of work station during data collection period), lack of resources and a limited number of hospitals included in the study, we were unable to obtain the optimum sample size.

### 2.4. Interviews and Data Collection

So as to obtain permission to collect the data, a letter was sent to the hospital administrator of each hospital explaining the purpose, implication and investigation process of the research. Following the administrative approval of the hospitals, research staff physically contacted the head of the kitchen or the dietary department in the study hospitals to know the rules of movement in the hospital areas, feasibility and appropriate time of data collection. Data were collected through face-to-face interviews using a structured questionnaire. Three interviewers, who were trained by the principal investigator of the study, visited the hospitals and were responsible for data collection. The lead investigator of the study arranged an online training session to train the data collectors about different sections of the questionnaire, interview techniques and the inclusion/exclusion criteria of the study. In terms of the food service staff of the patients’ kitchen, the interview was taken in the resting room of the kitchen as access to the kitchen was restricted. In the case of cafeteria food service staff, interviews were conducted in the cafeteria dining area where medical staff and visitors ate their meals. The interview was executed according to the convenient time of both kitchen and cafeteria food service staff so that their work might not be hampered. At first, trained data collectors explained the objectives of the study to the participants and asked for their voluntary participation. Written consent was obtained from those who were willing to participate in the survey (verbal consent was taken from those who were illiterate). Each interview took 12–15 min to accomplish. Unique identifier codes were assigned to the questionnaire to assure the anonymity of the participants.

### 2.5. Study Variables and Measures

Several published, reliable and validated questionnaires that had been used in similar studies [29,30,40,41] served as guides for developing the structured questionnaire. The questionnaire contained a total of 58 items of close-ended questions, divided into four parts, as follows: (i) sociodemographic and employment-related information (14 questions), (ii) assessment of food safety knowledge (18 questions), (iii) assessment of food safety attitude (11 questions) and (iv) assessment of food safety practices (15 questions).

#### 2.5.1. Explanatory Variables

Participants’ sociodemographic information, such as their age (years), gender, marital status, education status, employment type, monthly income, work experience (years), current employment position, food safety training status and idea about hazard analysis and critical control point (HACCP) system were included. Different characteristics relating to the hospitals, such as the employed hospital type, number of beds in the hospitals, working sector, type of hospital, the number of beds in the hospital and the person in charge of food service operations, were also collected.

#### 2.5.2. Outcome Measures

A set of 18 questions with three possible answers (“True”, “False” and “Don’t know”) was used to assess the participants’ knowledge regarding food safety. This section included information on personal hygiene and sanitation, cross-contamination, utensils cleaning, correct temperature, reheating of food and foodborne pathogens and illness. According to Banna et al. [34] and Bou-Mitri et al. [29], each correct answer that was addressed as “True” was marked as 1 point, while “False” and “Don’t know” responses were given zero points in this section, except question number five. For question number “5”, 1 point was attributed for “False” while zero points were given for “True” and “Don’t know” responses. Each participant could obtain a total score between 0 and 18, and a higher score indicated a higher level of food safety knowledge.

Participants’ attitudes toward food safety were assessed by an 11-items scale. This section included information on responsibility for food safety measures, the impact of foodborne illness on socio-economic developments, food contamination and maintenance of refrigerators. All questions had three possible answers: “agree”, “disagree” and “no idea”. For each response of “agree”, 1 point was given, whereas for responses of “disagree” and “no idea”, the score was 0 [29]. Summing up the total scores (range: 0–11) contributed to gathering estimation of the food safety attitudes of a participant.

Food safety practices included information on the role of personal hygiene, hand-washing practices and strategies against foodborne diseases and cross-contamination. A list of 15 questions was provided, and practices were evaluated on an ordinal scale of 0 to 4 (Never = 0, Rarely = 1, Sometimes = 2, Often = 3, Always = 4). Only 4 questions were scored in reverse (items 9, 10, 11 and 15) where “Never”, “Rarely”, “Sometimes”, “Often” and “Always” were considered as “4”, “3”, “2”, “1” and “0” points, respectively. The calculated total score of food safety practices ranged from 0 to 60, and a higher score indicated a good level of practice [34].

### 2.6. Validity and Reliability of the Questionnaire

The translation back-translation technique was used to check the content validity of the questionnaire [31]. The questionnaire was translated from English to Bengali (native language) by a bilingual translator, which was checked by an independent research assistant. Back-translation of the questionnaire was conducted by a separate independent bilingual research assistant to check for consistency and to avoid any bias in the questionnaire. Questionnaire reliability, the degree to which the food safety knowledge, attitudes and practices test was consistent, was measured using a pilot survey and internal consistency of the questionnaire. The questionnaire was pre-tested among a small sample of food service staff from the hospital cafeteria (n = 10) to ensure that there were no ambiguous or unclear questions. However, the results from the pilot survey were not included in the final analysis. The internal consistency (Cronbach’s alpha, α) for the food safety knowledge, attitudes and practice sections was 0.79, 0.71 and 0.73, respectively, indicating an acceptable level of reliability.

### 2.7. Statistical Analyses

Descriptive statistics such as frequency, percentage, mean and standard deviations were computed to summarize variables of interest. A one-way analysis of variance (ANOVA) and independent sample *t*-test were used to compare food safety knowledge scores across the independent variables (normally distributed). When data follow a skewed distribution, non-parametric analysis is the most appropriate method for analyzing the data. Kruskal–Wallis tests or Wilcoxon rank-sum tests were used to compare food safety attitudes and practices scores across their sociodemographic and employment characteristics. A multiple linear model was used to identify the factors associated with high scores of food safety knowledge, attitudes and practices. All assumptions were checked regarding linear regression after fitting the model. Regression coefficients (β) with 95% CI were used to quantify associations. All statistical analyses were done using Stata (BE version 17.0, StataCorp, College Station, TX, USA) and SPSS (IBM version 23.0, Armonk, NY, USA), and statistical significance was set at *p* < 0.05.

## 3. Results

### 3.1. Sociodemographic and Employment-Related Characteristics of Study Participants (N = 191)

Of the 191 participants, most of them (90.6%) were male. The mean age of the participants was 26.8 years (SD = 7.2), ranging between 18 and 50 years. Nearly half of the participants (46.1%) had a primary level of education, and about 20% had no formal education. Almost two-thirds of the participants’ (61%) monthly income was 10,000 to 20,000 Bangladeshi taka, BDT (111.63 to 223.26 USD). Only 18% of the employees served more than 10 years, while most were full-time employees (81.7%). Almost half of the respondents (43.5%) did not receive any training on food safety, and most of the respondents (76.4%) had no idea about HACCP. A majority (85%) of the employees were from private hospitals, where 40.8% worked in the patient meals sector (Table 1).

### 3.2. Food Safety Knowledge and Its Associated Factors among Food Handlers in Bangladeshi Hospitals

An assessment of food safety knowledge of food handlers working at hospitals is summarized in Table 2. The majority of the participants reported high knowledge of cross-contamination (87.4%), proper cleaning procedures of the equipment (94.8%), vulnerable groups for food poisoning (76.4%) and disease transmission such as wearing gloves while handling food reduced the risk of transmitting an infection to consumers (88.5%) and food service staff (86.4%) and necessary to take leave from work when affected by infectious diseases (87.4%). However, most of the participants showed lower knowledge regarding selected foodborne diseases (except for diarrhea) and foodborne pathogens (Table 2).

The mean food safety knowledge score was 10.75 on a scale of 18.0 (SD = 3.08, range: 3–18), and the overall correct response rate for the knowledge test was 59.7% (10.75/18 × 100), indicating moderate knowledge. The mean score of knowledge regarding food safety was varied significantly (all *p* < 0.05) by all the sociodemographic and employment-related characteristics of participants, except the participants’ age and marital status (Table 5).

The adjusted multiple linear analysis demonstrated that the knowledge score regarding food safety was significantly higher among male respondents compared to the females (β = 1.82, *p* < 0.05). Participants from private hospitals had higher knowledge of food safety than those from government hospitals (β = 4.00, *p* < 0.05). Compared to participants working in the patients’ meal sector, those who worked in the cafeteria were more knowledgeable (β = 4.69, *p* < 0.05). Similarly, participants working in a hospital that had a food service supervisor (β = 4.36, *p* < 0.05) and dietitian (β = 5.82, *p* < 0.05) in charge of food service operations had more knowledge compared to those from hospitals having a hospital administrator in charge (Table 6).

### 3.3. Food Safety Attitudes and Associated Factors among Food Handlers in Bangladeshi Hospitals

An assessment of food safety attitudes of food handlers working at hospitals is presented in Table 3. The majority of the respondents reported positive attitudes toward the responsibility for food safety measures (95.8%), food storage such as storing raw and cooked food separately (92.7%), food hygiene training (90.1%) and checking temperatures of refrigerators (80.6%). However, 41.9% and 60.7% of the respondents either disagreed or did not have any idea about the refrigeration of leftover foods and unsafe practice of leaving cooked food out of the refrigerator for more than two hours, respectively (Table 3).

The average food safety attitude score was 8.32 (SD = 1.96, range: 3–11) out of 11.0, and the overall correct response rate for the attitude test was 75.6% (8.32/11 × 100), indicating a higher level of positive attitudes. The average score of attitudes toward food safety significantly differed based on respondents’ work experience duration, employment type, employment position, receiving training on food safety, ideas about the HACCP system, hospital type, working sector and hospitals being in charge of food service operations (all *p* < 0.005) (Table 5).

The adjusted linear regression analysis showed that participants from private hospitals had a higher level of positive attitudes toward food safety than those from government hospitals (β = 2.29, *p* < 0.05). Those who worked in the cafeteria were more positive attitudes compared to participants working in the patients’ meal sector (β = 2.84, *p* < 0.05). Participants working in a hospital that had a food service supervisor (β = 2.11, *p* < 0.05) and dietitian (β = 2.37, *p* < 0.05) in charge of food service operations had more positive attitudes compared to those from hospitals having a hospital administrator in charge (Table 6).

### 3.4. Food Safety Practices and Associated Factors among Food Handlers in Hospitals

An evaluation of food safety practices of food handlers working at hospitals is shown in Table 4. The majority of the respondents reported that they always washed their hands before and after touching unwrapped raw and cooked food (70.2–89.5%) and maintained personal hygiene practices at workplaces such as wearing apron (70.7%), using hair cover (72.3%) and mask (77.5%) (Table 4).

The average score for food safety practices was 50.09 (SD = 9.58, range: 26–60) on a scale of 60.0, and the overall correct response rate for the practice test was 83.5% (50.09/60 × 100), indicating a higher level of practices. As shown in Table 5, the average score of practice was significantly varied by participants’ educational level, monthly income, work experience duration, employment position, receiving training on food safety, ideas about the HACCP system, hospital type, working sector and hospitals being in charge of food service operations (all *p* < 0.005) (Table 5).

Participants from private hospitals had higher practice scores regarding food safety compared to those from government hospitals (β = 15.93, *p* < 0.05). Participants working in a hospital that had a nutritionist (β = 5.15, *p* < 0.05), food service supervisor (β = 11.44, *p* < 0.05) and dietitian (β = 13.01, *p* < 0.05) in charge of food service operations had better food safety practice compared to those from hospitals that had a hospital administrator in charge (Table 6).

### 3.5. Association of Food Safety Knowledge, Attitudes and Practice Scores

The adjusted regression models showed that participants’ food safety knowledge was positively associated with their attitude (β = 0.18, *p* < 0.05) but not with their practice (β = 0.31, *p* > 0.05). On the other hand, participants’ attitude scores were positively associated with their food safety practice scores (β = 0.87, *p* < 0.05) (Table 6).

## 4. Discussion

The current study assessed food safety knowledge, attitudes and practices of food service staff in Bangladeshi hospitals. Overall, this study found moderate knowledge among food handlers in the hospitals. The findings are consistent with previous studies in Malaysia [28], Sudan [30], Jordan [31], Saudi Arabia [33] and South Africa [32]. However, the findings differed from the study in Sri Lanka [27], which found that hospital food workers lacked basic food safety knowledge. It is critical to emphasize the importance of seeking continual improvement in the food service industry. These findings indicate the need for food handlers’ knowledge of food safety to be improved [10].

Specifically, the majority of the food service staff in the present study had poor knowledge of foodborne pathogens such as *E. coli*, *Salmonella* sp., *Staphylococcus* sp. and *Clostridium botulinum* and foodborne diseases such as hepatitis A and typhoid fever [except diarrhea]. These findings are similar to previous studies among hospital food service staff in Jordan [31] and Lebanon [29]. However, the majority of the respondents reported diarrhea as a symptom of foodborne illness; this was because it had been the most frequently occurring foodborne disease in Bangladesh [15]. Therefore, this study suggests the need for educational programs on food safety among hospital food handlers that include the causes of foodborne illnesses occurrence, pathogens associated with those illnesses, proper control measures and food handling practices, which can help to reduce foodborne disease outbreaks.

The mean score of knowledge regarding food safety varied significantly across sociodemographic characteristics. This is similar to other previous studies [33,34] on food safety knowledge. Specifically, the study showed that males had a significantly higher knowledge score compared to females. Sanlier and Konaklioglu reported a similar finding among males and females as far as food safety knowledge was concerned [42]. However, Patil et al. reported that men were found with poor food safety practices [43], while Alqurashi et al. in Saudi Arabia found that females had better food safety knowledge [33]. Despite this, Mclntyre et al. and Lee et al. did not find any association between gender and food safety knowledge [10,44]. The possible reasons for the variations in findings could be differences in study populations, setting and cultural variations.

As found in previous studies in Jordan [31] and Iran [45], this current study indicated that participants from private hospitals had higher knowledge, attitudes and practices scores on food safety than those from government hospitals. Begum et al. mentioned food service as one of the more emergent supporting services compared to other hospital services [23]. Usually, government hospitals have scarce funding, governance issues and a limited skilled workforce, although the Government of Bangladesh has been working hard to fill the gaps in the national healthcare system [23,46,47,48]. Another possible reason could be that most of the well-established private hospitals are located in the capital and divisional cities of Bangladesh. In addition, clients in private hospitals are monitored and subjected to regular refresher training on food safety compared to those in government hospitals, as explained by Osaili et al. [31].

Moreover, participants working in a hospital that had a food service supervisor and dietitian in charge of food service operations had more knowledge and had a more positive attitude toward food safety compared to those from hospitals having a hospital administrator in charge. Gruenfeldova et al. argued that guidance and supervision, in addition to training, are required for improved practices as training is not necessarily related to practices [49]. In addition, those who are supervised by food service supervisors, dietitians and nutritionists are usually given on-the-job training because of their knowledge of food safety practices [31]. This calls for the use of food service supervisors, dieticians and nutritionists who are well vexed in food management and safety to supervise food handlers to improve their knowledge and attitudes toward food safety.

Food handlers’ attitudes can influence their food safety behavior and practice, which can help to minimize the spread of foodborne diseases and other health conditions [50]. In terms of food safety attitudes, the study showed that the respondents had a higher level of positive attitudes toward food safety. This is consistent with previous studies in Bangladesh [50] and Sudan [30]. In terms of food safety practices, there was a higher level of practices among respondents (for example, the majority of them always washed their hands before and after touching unwrapped raw and cooked food). This is consistent with several previous studies in Indonesia [51], Malaysia [52], Jordan [53] and Bangladesh [50]. The study further showed that respondents with high monthly incomes were more likely to practice good food hygiene. Our finding confirms other studies in Ethiopia [54], Ghana [55] and Jordan [31]. The possible explanation is that those with high monthly income may be able to acquire additional materials needed to establish themselves in hygienic conditions [such as personal protective equipment, hand sanitizer] and acquire extra cooking training, including food safety practices [34]. In Bangladesh, hospital authorities generally provide PPE to staff; however, the quality and adequacy of PPE can be a concern, especially during the crisis period [56]. A recent study reported that nearly half of the respondents [50%] claimed that there was an inadequate supply of PPE for hospitals and healthcare workers [56]. Hence, financial solvency enables them to carry hygienic materials for their own use regardless of their workplace.

Consistent with previous studies [57,58], educational level and receiving training on food safety were associated with good food safety practices. The probable explanation is that those with a higher level of education can understand some of the regulations and instructions pertaining to food safety practices. In this study, training appears to have been significant for knowledge, attitudes and practice; yet only about half of the sample received food safety training. Training programs are therefore important for improving the knowledge of food handlers [59] and can be recommended for all food service employees, regardless of educational level. The study further showed that those with the idea that the HACCP system had good food safety practices. This calls for the need to raise more awareness about the HACCP system through training.

### 4.1. Strength and Limitations

Several limitations should be considered when interpreting the findings of this study. First, the study employed a cross-sectional study design; therefore, the findings can only be interpreted in terms of associations but cannot claim causality among the variables studied. Second, this study was unable to obtain the calculated sample size due to the exclusion procedures, lack of resources and a limited number of hospitals included in the study. Third, the hospitals and participants included in this study were selected from two major divisional cities of Bangladesh (Dhaka and Chattogram), so the findings cannot be generalized across the country. Further studies, particularly incorporating food service staff from other districts and sub-district-level hospitals, are highly recommended. Fourth, the selection of the hospitals and participants were both done using a non-probability sampling technique which might affect the findings of this study. Fifth, there was a lack of factor analysis to see if the scale items represent the same construct. Sixth, the female sample is much smaller than the male group. Further studies that include more hospitals and large and diverse samples are warranted to yield concrete statistical comparisons between male and female food service employees. Moreover, there is the possibility of social desirability and reporting biases from the respondents due to the self-report nature of the measurements used in this study. Despite these limitations, there are several strengths to this study. This was one of the first studies to consider the knowledge, attitudes and practice of food safety among hospital food handlers in Bangladesh. The findings of this study provide a baseline resource for policymakers and public health practitioners to aid with the development and implementation of evidence-based interventions and initiatives to improve food safety knowledge, attitudes and practices among food service staff in Bangladesh. Additionally, this study has methodological and analytical rigor with detailed and reproducible approaches for future research in other similar settings.

### 4.2. Implication for Practice

Foodborne illness and food safety are still significant public health issues in Bangladesh [13,60]. As hospitals are more susceptible to potential hazards, food safety knowledge, attitudes and practices among hospital food service staff are crucial in the prevention of foodborne disease outbreaks. The findings of our study can contribute to the growing body of literature and provide preliminary information on food service staff’s knowledge, attitudes and practices regarding food safety in Bangladeshi hospitals to inform policies and future research. Specifically, our study found that participants from private hospitals and those who work in the hospitals that had a food service supervisor and dietitian in charge of food services had higher knowledge, attitudes and practices on food safety compared to their counterparts. These findings highlight a dire need to appoint dietitians and nutritionists in Bangladeshi hospitals, particularly in governmental hospitals where there is an absence of food and nutrition experts who are proficient enough in dietary management, food safety and hygiene.

## 5. Conclusions

This current study found moderate knowledge but high levels of attitudes and practices of food safety among hospital food service staff. The knowledge, attitudes and practices varied by demographic and work-related characteristics. To increase food safety knowledge, attitudes and practices, the associated factors should be taken into consideration. Specifically, hospital management should increase training and supervision on food safety practices to reduce foodborne diseases and outbreaks. Further studies could also employ qualitative designs to gain a deeper understanding of the enablers and barriers toward food safety practices.

## Figures and Tables

**Table 1:** Sociodemographic and employment characteristics of study participants (N = 191).

Characteristics	Frequency	Percentage
**Gender**		
Male	173	90.6
Female	18	9.4
**Age (in year)**		
18–25	94	49.2
26–35	70	36.6
≥36	27	14.2
**Marital status**		
Single	107	56.0
Married	84	44.0
**Education status**		
No formal education	36	18.8
Primary	88	46.1
Secondary	47	24.6
Higher Secondary	13	6.8
Honors or above	7	3.7
**Monthly income (in BDT)**		
<10,000	48	25.1
10,001–15,000	58	30.4
15,001–20,000	59	30.9
20,001–25,000	14	7.3
>25,000	12	6.3
**Work experience (in years)**		
<5	79	41.4
5–10	77	40.3
>10	35	18.3
**Employment type**		
Full time	156	81.7
Part time	21	11.0
Temporal	14	7.3
**Current employment position**		
Food service supervisor	9	4.7
Chef	71	37.2
Support staff	111	58.1
**Food safety training**		
Yes	108	56.5
No	83	43.5
**Idea about HACCP system**		
Yes	45	23.6
No	146	76.4
**Employed hospital type**		
Government	28	14.7
Private	163	85.3
**Number of beds in the hospital**		
<250	94	49.2
≥250	97	50.8
**Working sectors**		
Patient meals	78	40.8
Cafeteria for staff and visitors	113	59.2
**In charge of food service operation in the hospital**		
Hospital administrator	125	65.4
Nutritionist	8	4.2
Food service supervisor	34	17.8
Dietitian	24	12.6

**Table 2:** Summary of questions and responses for assessment of food safety knowledge of food handlers working at hospitals in Bangladesh (N = 191).

Statements	Responses, n (%)		
	True	False	Don’t Know
Preparation of food in advance could contribute to food poisoning.	167 (87.4)	14 (7.3)	10 (5.2)
Reheating food is likely to contribute to food contamination.	128 (67)	43 (22.5)	20 (10.5)
The correct temperature for storing perishable foods is 5 °C.	99 (51.8)	23 (12)	69 (36.1)
Incorrect application of cleaning/sanitization procedures on equipment (refrigerator, slicing machine) can increase the risk of foodborne disease to consumers.	181 (94.8)	6 (3.1)	4 (2.1)
Hand washing before handling food, using only water reduce the risk of contamination.	32 (16.8)	150 (78.5)	9 (4.7)
Wearing gloves while handling food reduces the risk of transmitting infection to consumers.	169 (88.5)	7 (3.7)	15 (7.9)
Wearing gloves while handling food reduces the risk of transmitting infection to food-services staff.	165 (86.4)	13 (6.8)	13 (6.8)
Eating and drinking in the work place increases the risk of food contamination.	114 (59.7)	54 (28.3)	23 (12)
During infectious disease of skin and eye, it is necessary to take leave from work.	167 (87.4)	13 (6.8)	11 (5.8)
Cross-contamination is when microorganisms from a contaminated food are transferred by the food handler’s hands or utensils to another.	167 (87.4)	8 (4.2)	16 (8.4)
All persons, including children, adults, pregnant women and old-ages are at equal risk for food poisoning.	146 (76.4)	27 (14.1)	18 (9.4)
Typhoid can be transmitted by food.	72 (37.7)	32 (16.8)	87 (45.5)
Diarrhea can be transmitted by food.	173 (90.6)	11 (5.8)	7 (3.7)
Hepatitis A can be transmitted by food.	53 (27.7)	43 (22.5)	95 (49.7)
*E. coli* is a food borne pathogen.	32 (16.8)	3 (1.6)	156 (81.7)
Salmonella is among the foodborne pathogens.	27 (14.1)	2 (1)	162 (84.8)
Staphylococcus is among the foodborne pathogens.	21 (11)	1 (0.5)	169 (88.5)
*Clostridium botulinum* is among the foodborne pathogens.	22 (11.5)	1 (0.5)	168 (88)

**Table 3:** Summary of questions and responses for assessment of food safety attitudes of food handlers working at hospitals in Bangladesh (N = 191).

Statements	Responses, n (%)		
	Agree	Disagree	No Idea
One of the most important responsibilities of the food handlers is to maintain food safety measures.	183 (95.8)	6 (3.1)	2 (1)
Raw and cooked foods should be stored separately to reduce risk of food contamination.	177 (92.7)	9 (4.7)	5 (2.6)
Food hygiene training for workers is an important issue in reducing risk of food contamination.	172 (90.1)	9 (4.7)	10 (5.2)
It is necessary to check the temperature of the refrigerator periodically to reduce risk of food contamination.	154 (80.6)	12 (6.3)	25 (13.1)
Health status of the workers should be evaluated before employment.	116 (60.7)	40 (20.9)	35 (18.3)
Foodborne illnesses can have deleterious health and economic effects on the society.	143 (74.9)	19 (9.9)	29 (15.2)
Safe food handling to avoid contamination and diseases is part of food-service staff’s job responsibilities.	166 (86.9)	16 (8.4)	9 (4.7)
After serving food, any leftovers should be kept in refrigerator.	111 (58.1)	63 (33)	17 (8.9)
I think it is unsafe to leave cooked food out of the refrigerator for more than two hours.	75 (39.3)	44 (23)	72 (37.7)
Using watches, earrings and rings will increase the risk of food contamination.	118 (61.8)	51 (26.7)	22 (11.5)
Food handlers who have abrasions or cuts on hands should not touch foods without gloves.	174 (91.1)	13 (6.8)	4 (2.1)

**Table 4:** Summary of questions and responses for assessment of food safety practices of food handlers working at hospitals in Bangladesh (N = 191).

Food Safety Practices	Responses, n (%)				
	Never	Rarely	Sometimes	Often	Always
Do you wash your hands before touching unwrapped raw food?	9 (4.7)	14 (7.3)	26 (13.6)	8 (4.2)	134 (70.2)
Do you wash your hands after touching unwrapped raw food?	5 (2.6)	9 (4.7)	25 (13.1)	4 (2.1)	148 (77.5)
Do you wash your hands before touching unwrapped cooked food?	1 (0.5)	2 (1)	21 (11)	7 (3.7)	160 (83.8)
Do you wash your hands after touching unwrapped cooked food?	1 (0.5)	2 (1)	12 (6.3)	5 (2.6)	171 (89.5)
Do you wear apron during work?	12 (6.3)	7 (3.7)	24 (12.6)	13 (6.8)	135 (70.7)
Do you use hair cover during work?	18 (9.4)	3 (1.6)	20 (10.5)	12 (6.3)	138 (72.3)
Do you use mask when you prepare or distribute foods?	6 (3.1)	5 (2.6)	16 (8.4)	16 (8.4)	148 (77.5)
Do you use separate kitchen utensils to prepare raw and cooked food?	16 (8.4)	4 (2.1)	14 (7.3)	7 (3.7)	150 (78.5)
If you have lesions on your hands, you treat yourself and complete your work?	118 (61.8)	9 (4.7)	14 (7.3)	36 (18.8)	14 (7.3)
Do you allow your finger nails to grow as wearing gloves protects the transmission of germs?	169 (88.5)	2 (1)	14 (7.3)	2 (1)	4 (2.1)
Do you thaw food at room temperature?	26 (13.6)	14 (7.3)	55 (28.8)	41 (21.5)	55 (28.8)
Do you ask for cleaning food contact surfaces before and after preparing food?	4 (2.1)	2 (1)	25 (13.1)	7 (3.7)	153 (80.1)
Do you check shelf life of food products while using them?	6 (3.1)	5 (2.6)	25 (13.1)	9 (4.7)	146 (76.4)
Do you check integrity of food packages while buying food products?	17 (8.9)	5 (2.6)	30 (15.7)	15 (7.9)	124 (64.9)
Do you smoke inside food processing areas?	172 (90.1)	2 (1)	14 (7.3)	2 (1)	1 (0.5)

**Table 5:** Respondents’ food safety knowledge, attitudes and practices by their sociodemographic and employment characteristics (N = 191).

Characteristics	Knowledge Score		Attitude Score		Practice Score	
	Mean ± SD	*p* Value ^†^	Mean ± SD	*p* Value ^††^	Mean ± SD	*p* Value ^††^
**Gender**		***0.003***		0.092		0.159
Male	10.96 ± 3.03		8.40 ± 1.94		50.16 ± 9.83	
Female	8.72 ± 2.82		7.56 ± 2.04		49.44 ± 6.91	
**Age (in year)**		0.576		0.579		0.533
18–25	10.57 ± 2.43		8.50 ± 1.80		51.66 ± 8.68	
26–35	11.06 ± 3.37		8.13 ± 2.01		49.21 ± 9.97	
36–45	10.56 ± 4.19		8.19 ± 2.35		46.89 ± 10.73	
**Marital status**		0.188		0.476		0.378
Single	10.48 ± 2.52		8.26 ± 1.90		50.02 ± 9.79	
Married	11.10 ± 3.66		8.39 ± 2.04		50.18 ± 9.36	
**Education status**		***<0.001***		0.062		***0.006***
No formal education	10.06 ± 2.76		8.56 ± 2.01		52.17 ± 7.57	
Primary	10.33 ± 2.53		8.07 ± 1.90		49.62 ± 9.81	
Secondary	10.66 ± 3.05		8.21 ± 2.19		47.49 ± 10.73	
Higher Secondary	12.62 ± 4.21		8.92 ± 1.32		53.31 ± 8.26	
Honors or above	16.71 ± 1.11		9.86 ± 0.69		56.71 ± 0.76	
**Monthly income (in BDT)**		***<0.001***		0.051		***<0.001***
<10,000	9.90 ± 2.68		7.94 ± 2.16		48.35 ± 9.09	
10,001–15,000	10.26 ± 2.75		8.34 ± 2.05		51.53 ± 9.10	
15,001–20,000	11.08 ± 2.57		8.41 ± 1.74		50.69 ± 9.51	
20,001–25,000	9.71 ± 3.85		7.93 ± 2.06		41.93 ± 11.93	
>25,000	16.08 ± 1.93		9.75 ± 0.62		56.58 ± 0.90	
**Work experience (in years)**		***<0.001***		***0.031***		***0.003***
<5	9.78 ± 2.54		7.87 ± 2.05		47.94 ± 10.02	
5–10	11.30 ± 3.12		8.62 ± 1.75		52.18 ± 8.21	
>10	11.71 ± 3.56		8.66 ± 2.04		50.34 ± 10.53	
**Employment type**		***0.011***		***0.045***		0.054
Full time	11.00 ± 3.06		8.45 ± 1.85		50.67 ± 9.38	
Part time	8.86 ± 2.29		7.10 ± 2.47		44.29 ± 10.76	
Temporal	10.79 ± 3.45		8.71 ± 1.68		52.36 ± 7.09	
**Employment position**		***<0.001***		***0.023***		***0.042***
Food service supervisor	15.33 ± 3.54		9.56 ± 1.42		54.78 ± 5.22	
Chef	10.13 ± 2.89		7.94 ± 2.01		47.34 ± 10.93	
Support staff	10.77 ± 2.85		8.46 ± 1.91		51.47 ± 8.47	
**Food safety training**		***<0.001***		***<0.001***		***<0.001***
Yes	12.13 ± 2.78		9.15 ± 1.34		54.81 ± 4.82	
No	8.95 ± 2.46		7.24 ± 2.11		43.94 ± 10.70	
**Idea about HACCP system**		***<0.001***		***0.006***		***<0.001***
Yes	12.82 ± 3.39		9.09 ± 1.28		53.58 ± 6.97	
No	10.11 ± 2.68		8.08 ± 2.07		49.01 ± 10.02	
**Employed hospital type**		***<0.001***		***<0.001***		***<0.001***
Government	7.86 ± 1.96		6.07 ± 1.27		35.50 ± 6.36	
Private	11.25 ± 2.96		8.71 ± 1.79		52.60 ± 7.60	
**Number of beds in the hospital**		***0.019***		0.194		0.132
<250	11.28 ± 3.16		8.47 ± 1.99		50.90 ± 9.24	
>250	10.24 ± 2.92		8.18 ± 1.92		49.30 ± 9.88	
**Working sectors**		***<0.001***		***<0.001***		***<0.001***
Patient meals	9.46 ± 3.31		7.10 ± 2.04		42.64 ± 10.92	
Cafeteria for staff and visitors	11.64 ± 2.58		9.16 ± 1.38		55.23 ± 2.89	
**In charge of food service operation in the hospital**		***<0.001***		***<0.001***		***<0.001***
Hospital administrator	11.18 ± 2.86		8.86 ± 1.70		53.36 ± 6.54	
Nutritionist	7.88 ± 2.53		5.63 ± 0.92		37.50 ± 7.11	
Food service supervisor Dietitian	11.71 ± 3.29 8.08 ± 1.98		8.53 ± 1.90 6.13 ± 1.23		50.91 ± 10.22 36.08 ± 6.53	

Note: ^†^ *p*-value was determined by one-way ANOVA or independent sample *t*-test. ^††^ *p*-value was determined by Kruskal–Wallis test or Wilcoxon Rank Sum Test. Bolded and italic values indicates statistically significant (*p* < 0.05).

**Table 6:** Multiple linear regression models identifying the factors associated with food safety knowledge, attitudes and practices among study participants (N = 191).

Variables	Model 1: Food Safety Knowledge		Model 2: Food Safety Attitudes		Model 3: Food Safety Practices	
	β	95% CI	β	95% CI	β	95% CI
**Gender**						
Male	1.82 *	0.53, 3.10	0.49	−0.36, 1.35	−0.66	−3.43, 2.11
Female	RC		RC		RC	
**Age (in year)**						
18–25	RC		RC		RC	
26–35	0.37	−0.63, 1.37	−0.02	−0.68, 0.63	−0.30	−2.40, 1.80
36–45	−0.52	−2.22, 1.08	0.60	−0.44, 1.64	1.03	−2.33, 4.39
**Marital status**						
Single	RC		RC		RC	
Married	0.23	−0.80, 1.26	−0.04	−0.71, 0.63	0.14	−2.01, 2.29
**Education status**						
No formal education	RC		RC		RC	
Primary	0.19	−0.70, 1.07	−0.39	−0.97, 0.18	−0.63	−2.48, 1.23
Secondary	0.37	−0.68, 1.42	−0.00	−0.68, 0.68	−1.42	−3.62, 0.78
Higher Secondary	0.70	−0.86, 2.27	−0.27	−1.29, 0.75	0.69	−2.60, 3.97
Honors or above	2.25	−0.59, 5.10	−0.54	−2.40, 1.32	0.86	−5.14, 6.87
**Monthly Income (BDT)**						
<10,000	RC		RC		RC	
10,001–15,000	−0.03	−0.96, 0.90	0.20	−0.40, 0.80	2.82 *	0.87, 4.77
15,001–20,000	0.84	−0.39, 2.06	0.68	−0.13, 1.48	5.07 *	2.47, 7.67
20,001–25,000	1.19	−0.64, 3.02	1.39 *	0.20, 2.59	1.22	−2.69, 5.13
>25,000	3.19 *	0.84, 5.55	0.13	−1.44, 1.69	2.43	−2.61, 7.48
**Work experience**						
<5 years	RC		RC		RC	
5–10 years	0.43	−0.38, 2.05	0.13	−0.40, 0.66	0.91	−0.79, 2.62
>10 years	0.45	−0.77, 1.66	0.09	−0.70, 0.88	−0.59	−3.14, 1.95
**Employment type**						
Full time	RC		RC		RC	
Part time	0.39	−0.72, 1.50	0.09	−0.63, 0.82	1.80	−0.53, 4.12
temporal	0.19	−1.03, 1.41	0.09	−0.71, 0.87	0.05	−2.51, 2.62
**Employment position**						
Food service supervisor	RC		RC		RC	
Chef	−0.51	−2.90, 1.88	−0.34	−1.90, 1.22	0.77	−4.24, 5.79
Support staff	0.14	−2.25, 2.54	−0.16	−1.72, 1.40	1.01	−4.01, 6.03
**Food safety training**						
Yes	0.75	−0.18, 1.68	0.12	−0.49, 0.73	1.61	−0.36, 3.57
No	RC		RC		RC	
**Idea about HACCP system**						
Yes	RC		RC		RC	
No	−0.26	−1.18, 0.65	0.27	−0.33, 0.87	0.69	−1.24, 2.62
**Employed hospital type**						
Government	RC		RC		RC 15.93 *	10.15, 21.71
Private	4.00 *	1.37, 6.64	2.29 *	0.52, 4.05		
**Number of beds in the hospital**						
<250	RC		RC		RC	
≥ 250	−0.26	−1.16, 0.64	0.14	−0.44, 0.73	0.94	−0.95, 2.82
**Working sectors**						
Patient meals	RC		RC		RC	
Cafeteria	4.69 *	2.99, 6.39	2.84 *	1.63, 4.04	13.72	9.60, 17.84
**In charge of food service operation in the hospital**						
Hospital administrator	RC		RC		RC	
Nutritionist	1.83	−0.42, 4.08	0.33	−1.14, 1.81	5.15 *	0.39, 9.90
Food service supervisor	4.36 *	2.63, 6.08	2.11 *	0.91, 3.32	11.44 *	7.42, 15.47
Dietitian	5.82 *	2.71, 8.93	2.37 *	0.26, 4.47	13.01 *	6.12, 19.89
**Food safety knowledge score**	NI		0.18 *	0.09, 0.29	0.31	−0.03, 0.64
**Food safety attitudes score**	NI		NI		0.87 *	0.37, 1.37
**Food safety practices score**	NI		NI		NI	

Note: CI = Confidence Interval, β = Regression coefficient, RC = Reference category, NI = Not included. The R^2^ for adjusted model 1, 2 and 3 was 0.8299, 0.6036 and 0.6198, respectively. Bolded and asterisk indicates statistically significant (*p* < 0.05).

## Data Availability

Data used in this analysis can be obtained by contacting the first author.
